# Efficacy of integrated traditional Chinese and western medicine in managing mild-moderate acute pancreatitis: a real-world clinical perspective analysis

**DOI:** 10.3389/fmed.2024.1429546

**Published:** 2024-10-30

**Authors:** Sailei Jia, Qian Chen, Xitong Liu, Yanhong Li, Lihui Wang, Xian Li, Shixiang Hu

**Affiliations:** ^1^Department of Critical Care Medicine, Henan Provincial Hospital of Traditional Chinese Medicine, Henan University of Traditional Chinese Medicine, Zhengzhou, China; ^2^Department of Critical Care Medicine, Henan Provincial Hospital of Traditional Chinese Medicine, The Second Affiliated Hospital of Henan University of Traditional Chinese Medicine, Zhengzhou, China; ^3^Department of Medical School, Huanghe Science and Technology College, Zhengzhou, China; ^4^Department of Gastroenterology, Henan Provincial Hospital of Traditional Chinese Medicine, The Second Affiliated Hospital of Henan University of Traditional Chinese Medicine, Zhengzhou, China

**Keywords:** integrated traditional Chinese and western medicine, acute pancreatitis, real-word study, mild-moderate pancreatitis, herbal enema

## Abstract

**Background:**

Given the prevalent utilization of integrated traditional Chinese and western medicine (ITCWM) in the management of acute pancreatitis, the majority of studies have concentrated on severe cases, lacking robust evidence-based medical research. Real-world investigations can provide an objective assessment of the clinical effectiveness of combining traditional Chinese medicine with western medicine. Consequently, relying on real-world research, we intend to evaluate the clinical efficacy and safety of the combined approach in treating mild to moderate acute pancreatitis.

**Methods:**

A total of 563 AP patients from Henan Provincial Hospital of Traditional Chinese Medicine were collected from January 2019 to October 2023. A propensity score matching (PSM) analysis was conducted to evaluate the clinical efficacy of traditional Chinese medicine (TCM) in treating mild to moderate acute pancreatitis. Patients were divided into a control group (61 cases) and an integrated traditional Chinese and Western medicine (ITCWM) group (120 cases). To further assess the clinical efficacy of TCM enema in the treatment of mild to moderate acute pancreatitis, PSM analysis was conducted across three groups. The patients were categorized into a control group (*n* = 49), an oral TCM treatment group (OCM group, *n* = 274), and an oral TCM plus enema treatment group (OCM+E group, *n* = 131). Logistic regression was used to analyze factors after treatment in each group, and the Kaplan-Meier method compared symptom duration in each group.

**Results:**

Compared with the control group, the ITCWM group significantly decreased C-reactive protein (CRP, mg/L) (17.8 [1.2–59.5] vs. 8.0 [3.3–33.5], *P* = 0.022), shortened the duration of abdominal distension, abdominal pain, nausea and bitter taste symptoms (*P* < 0.05), and shortened the length of hospital stay (median 19.0 and 11.5 days, respectively, *P* = 0.001); Compared with the other two groups, the neutrophil percentage (NEUT%) was lower (74.1 vs. 61.9 vs. 59.5, *P* < 0.05) and serum prealbumin (PA, mg/L) was higher (116.0 vs. 184.4 vs. 220.0, *P* < 0.05), the length of hospitalization (days) was shortened (19.0 vs.12.0 vs.10.0, *P* < 0.05) in the OCM+E group.

**Conclusion:**

The combination of traditional Chinese medicine and modern medicine has been shown to effectively decrease inflammatory indicators in patients with mild to moderate acute pancreatitis, leading to a reduction in symptom duration and hospitalization period, as well as promoting disease recovery. Notably, the use of traditional Chinese medicine in conjunction with enema therapy yields more pronounced benefits.

## 1 Introduction

Acute Pancreatitis (AP), a common and emergent digestive disease, arises from pancreatic enzyme activation, characterized by pancreatic inflammation and caused by various etiologies. AP often progresses from localized damage to systemic organ dysfunction, known as severe acute pancreatitis (SAP) (1). The annual incidence of AP in high-income countries is approximately 34/100,000. The AP incidence has increased by 62.9% and AP-related mortality by 64.8% since 1990 (2, 3).

Although self-limiting in most patients, moderate even severe AP still occurs in approximately 20% of patients, characterized by (surrounding) pancreatic tissue necrosis and/or (multi-organ) failure and a mortality rate up to 20–40% (4, 5). Early control on mild and moderate AP can effectively reduce disease progression and facilitate patients’ recovery, thereby contributing to better outcomes in AP (6).

Oral or external application of Traditional Chinese medicine (TCM), along with enema, has shown significant benefits in treating AP (7–9). TCM demonstrates efficacy in reducing capillary permeability, inhibiting the production of inflammatory cytokines, and suppressing neutrophil activation, thereby mitigating pancreatic injury (7, 10). Furthermore, TCM offers benefits such as improving clinical symptoms, reducing medical costs, and enhancing patient satisfaction, all of which position it as a viable complementary and alternative therapy for AP (11, 12). Despite its primary use in severe AP (13, 14), there lacks robust evidence-based medical research on TCM in treating mild to moderate AP. Real-world studies, as opposed to rigorous randomized controlled trials, provide a more authentic reflection of the advantages of TCM. These studies offer innovative perspectives and methods for evaluating treatment efficacy through evidence-based approaches. Thus, our retrospective cohort study, conducted in real-world and approved by the clinical medical research ethics committee of Henan Province Hospital of Traditional Chinese Medicine (ethics batch number: 1480), investigated the efficacy of TCM in treating mild to moderate AP. The study employed PSM to balance clinical baseline data across the groups.

## 2 Materials and methods

### 2.1 Data sources

Data of patients diagnosed with AP, and hospitalized within 48 hours of symptom onset, were retrospectively collected from hospital information system (HIS) of Henan Province Hospital of Traditional Chinese Medicine, spanning from January 2019 to October 2023. Uniformity of diagnosis and pathological staging was ensured by the data standardization processing according to the criteria outlined in ‘American Gastroenterological Association Institute Guideline on Initial Management of Acute pancreatitis’ (6).

### 2.2 Diagnostic criteria

The diagnostic criteria of AP followed the guidelines published in ‘American Gastroenterological Association Institute Guideline on Initial Management of Acute pancreatitis’ (6). The severity of acute pancreatitis was graded using the revised RAC score, based on the ‘Classification of Acute Pancreatitis—2012: Revision of the Atlanta Classification and Definitions by International Consensus’ (1). The diagnostic criteria of TCM symptoms or signs of AP were diagnosed based on the ‘The consensus of integrative diagnosis and treatment of acute pancreatitis-2017’ (15), which are divided into four syndrome types, namely, liver depression and qi stagnation, liver and gallbladder dampness and heat, chest binding and interior excess, blood stasis (toxin) binding with hot, see Supplementary Table 1 for the specific diagnosis.

### 2.3 Inclusion criteria

Eligible participants included: (1) admission within 48 hours of symptom onset; (2) age between 18 and 75 years; (3) informed consent, either from relatives or guardians, for voluntary participation in this study.

### 2.4 Exclusion criteria

Exclusion criteria included: (1) patients with severe AP requiring surgery or non-internal medicine treatments (abdominal lavage or others); (2) patients with severe hypertension (systolic blood pressure > 180 mmHg and limited response to medicines); (3) patients diagnosed with advanced-stage tumors; (4) patients had received other clinical trials within three months prior to disease onset; (5) pregnant or lactating women.

## 3 Methods

### 3.1 Grouping and treatment methods

**FIGURE 1:**
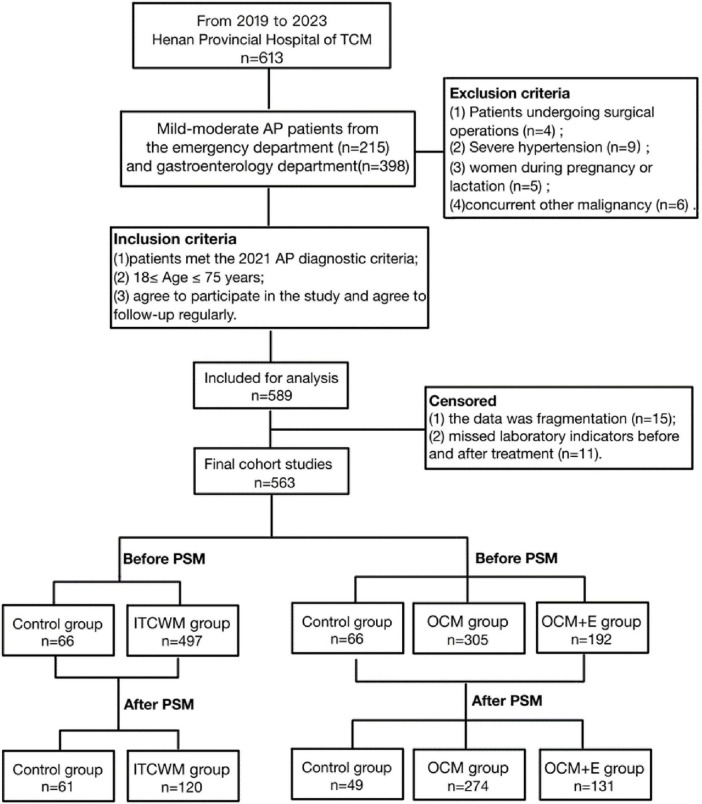
Flow diagram of the trial. TCM, Traditional Chinese Medicine; PSM, Propensity Score Matching; ITCWM group, Integrated Traditional Chinese and Western Medicine; OCM group, the Oral Chinese Medicine Treatment group; OCM+E group, the Oral Chinese Medicine and Enema treatment group.

Firstly, according to the treatment situation, it can be divided into two groups: the Control group and the Integrated Traditional Chinese and Western Medicine treatment group (the ITCWM group). The control group implemented contemporary medical treatment approaches, encompassing etiological treatment, fasting, early fluid resuscitation, analgesia, inhibition of digestive enzyme secretion, and nutritional support, among others. In contrast, the ITCWM group employed contemporary medical treatment methods in conjunction with TCM classification treatment, as outlined in Supplementary Table 1. In order to enhance the understanding of the clinical effectiveness of enema with traditional Chinese medicine, the treatment group consisting of integrated traditional Chinese and western medicine was divided into two subgroups: the simple oral Chinese medicine treatment group (OCM group) and the oral Chinese medicine and enema treatment group (OCM+E group). These two groups were then compared with the control group. Traditional Chinese medicine enema therapy involves making a mixture of 30g of raw rhubarb and 200mL of water. This mixture is then boiled, filtered to remove any residues, and cooled to a temperature of 38–40°C. The enema is administered with an intubation depth of 30–35 cm and should be retained for 1–2 h, twice a day. The study design is briefly described in Figure 1.

### 3.2 Propensity score matching

To mitigate the impact of potential confounding variables, we employed the MatchIt package in R software (version 4.2.2) to conduct bias matching between the two groups (16). A logistic regression model was conducted with TCM therapy as the dependent variable, and potential confounders as independent variables. The confounders were those that were AP-related and showed imbalances between the ITCWM group and the control group. With a caliper width set at a quarter of the log standard deviation of the propensity score, PSM for both two groups were performed utilizing the nearest neighbor matching (1:2), involving variables such as age, gender, smoking and alcohol use histories, past history, pre-treatment evaluative scores and laboratory. Matching quality was evaluated by comparing baseline characteristics between patients receiving ITCWM group and matched control patients. DECISIONLINNC 1.0 software^1^ was used to conduct three groups of propensity matching scores (PSM). The software employs the twang package’s mnps function to estimate the average processing effect for the entire population by comparing each sample’s trend score. It calculates the average weight of these scores and the sample count per group. The data is then divided into three groups, sorted by weight from largest to smallest. We created a data set by extracting and merging the highest-weighted samples from each group. To assess match quality, we conducted a comparative analysis of the standardized mean differences across the three sets of covariates and observed that the baseline characteristics were approximately balanced following the matching process.

### 3.3 Observation outcomes

Primary outcomes were shortenings in symptom durations (abdominal distension, abdominal pain, Constipation, nausea, and etc.) and improvements in key laboratory indicators (white blood cell count, neutrophil count, CRP levels, Procalcitonin levels) pre- and post-treatment. Secondary outcomes included various scores (BISAP score, MCTSI score, APACHE II score and etc.) and hospitalization duration in AP patients.

### 3.4 Statistical methods

Statistical analyses were executed using SPSS (version 26.0). Quantitative data were expressed as mean ± standard deviation, while qualitative data as frequency. The t-test was utilized for normally distributed data with homoscedasticity, whereas the rank sum test for data exhibiting non-normally distributed or heteroscedasticity. Counting data analysis was performed using adjusted Chi-squared test and rank sum test for ranked data. Logistic regression analysis was applied to analyze the clinical efficacy of each group, and Kaplan-Meier (K-M) analysis was used to determine the symptom relief duration. K-M survival curves were plotted using GraphPad Prism 8 software. A p-value of less than 0.05 was considered statistically significant.

## 4 Results

### 4.1 Clinical characteristics before and after PSM

From January 2019 to October 2023, we collected medical records of 613 AP patients at Henan Province Hospital of Traditional Chinese Medicine, of which 563 met the inclusion and exclusion criteria. Ultimately, 181 patients were enrolled after PSM, and divided into the control group and the ITCWM group. Analysis of clinical data pre- and post-PSM covered variables including age, gender, smoking and alcohol use histories, past history, pre-treatment evaluative scores and laboratory indicators. Before PSM comparison, the levels of serum amylase (SA) and serum lipase (SL) in each group were as follows: the SA level (U/L) (133.0 [69.0,349.0] in the ITCWM group vs. 156.5 [88.0,365.0] in the control group, *P* = 0.478), and SL level (U/L) (499.0 [112.0,1349.0] in the ITCWM group vs. 585.0 [118.0,1347.0] in the control group, *P* = 0.736). In addition, initial pre-PSM comparison revealed significant differences between two groups in urea (UREA) levels (mmol/L) (4.4 [3.5,5.5] in the ITCWM group vs. 4.6 [4.0,5.9] in the control group, *P* = 0.041), CRP level (mg/L) (15.0 [5.0,54.0]in the ITCWM group vs. 6.7 [2.0,32.0] in the control group, *P* = 0.004), and urinary amylase (UA) level (U/L) (744.0 [336.0,1843.0] in the ITCWM group vs. 503.0 [243.0,1066.0] in the control group, *P* = 0.014). However, after PSM, baseline characteristics were balanced (*P* > 0.05), and no significant differences were observed in symptom frequency between two groups (Table 1 and Supplementary Table 2).

**TABLE 1:** Comparison of basic characteristics between two groups before and after PSM.

Items (pre-treatment)	Before PSM			After PSM		
	Control group (66)	ITCWM group (497)	*P*	Control group (61)	ITCWM group (120)	*P*
Age (years)	40.0 [32.0, 54.0]	40.0 [34.0, 49.0]	0.976	38.0 [30.0, 49.0]	41.0 [35.0, 57.5]	0.326
Gender male [n (%)]	43 (65.2)	357 (71.8)	0.327	39 (63.9)	81 (67.5)	0.754
Weight	71.4 ± 13.1	72.0 ± 13.7	0.129	71.4 ± 13.1	72.0 ± 13.7	0.651
**Pathogenesis [n (%)]**						
Biliary	20 (30.3)	168 (33.8)	0.669	18 (29.5)	39 (32.5)	0.810
Lipogenic	36 (54.5)	331 (66.6)	0.073	35 (57.4)	70 (58.3)	1.000
Alcoholic	1 (1.5)	5 (1.0)	0.528	1 (1.6)	4 (3.3)	0.664
Smoke	17 (25.8)	126 (25.4)	1.000	15 (24.6)	30 (25.0)	1.000
Drink	19 (28.8)	151 (30.4)	0.903	17 (27.9)	32 (26.7)	1.000
**Co-morbidities [n (%)]**						
Hypertension	10 (15.2)	108 (21.7)	0.283	8 (13.1)	17 (14.2)	1.000
Diabetes	12 (18.2)	104 (20.9)	0.722	10 (16.4)	21 (17.5)	1.000
RAC (mild) [*n* (%)]	38 (58.5)	354 (71.1)	0.066	38 (58.5)	71 (54.6)	0.637
BISAP (points)	0.0 [0.0, 1.0]	0.0 [0.0, 1.0]	0.889	0.0 [0.0, 1.0]	0.0 [0.0, 1.0]	0.838
MCTSI (points)	2.0 [2.0, 2.0]	2.0 [2.0, 2.0]	0.998	2.0 [2.0, 2.0]	2.0 [2.0, 2.0]	0.752
APACHE II (points)	13.2 ± 2.6	13.1 ± 2.6	0.548	13.2 ± 2.6	13.1 ± 2.6	0.744
SOFA (points)	0.0 [0.0, 1.0]	0.0 [0.0, 1.0]	0.613	0.0 [0.0, 1.0]	0.0 [0.0, 1.0]	0.983
WBC (× 10^9^/L)	9.3 [5.7, 13.4]	9.8 [6.5, 13.0]	0.498	9.4 [5.7, 13.4]	9.8 [6.0, 12.7]	0.851
NEUT (%)	77.5 ± 13.1	76.9 ± 12.3	0.752	77.5 ± 13.1	76.9 ± 12.3	0.659
RBC (× 10^12^ /L)	4.6 ± 0.7	4.7 ± 0.7	0.728	4.6 ± 0.7	4.7 ± 0.7	0.789
HGB (g/L)	142.8 ± 21.9	142.1 ± 24.9	0.342	142.8 ± 21.9	142.1 ± 24.9	0.878
Ca (mmol/L)	2.3 ± 0.2	2.3 ± 0.2	0.052	2.3 ± 0.2	2.3 ± 0.2	0.896
PCT (ng/mL)	0.1 [0.1, 0.2]	0.1 [0.1, 0.2]	0.916	0.1 [0.1, 0.2]	0.1 [0.1, 0.1]	0.571
CRP (mg/L)	6.7 [2.0, 32.0]	15.0 [5.0, 54.0]	0.004	6.0 [1.5, 32.0]	11.0 [5.0, 32.0]	0.067
ALT (U/L)	28.5 [17.0, 75.0]	31.0 [17.0, 65.0]	0.704	28.0 [17.0, 75.0]	26.5 [17.0, 60.5]	0.457
AST (U/L)	24.5 [18.0, 51.0]	27.0 [20.0, 47.0]	0.331	24.0 [18.0, 49.0]	26.0 [19.0, 43.5]	0.987
ALB (g/L)	41.7 ± 5.7	41.2 ± 5.3	0.091	41.7 ± 5.7	41.2 ± 5.3	0.811
UREA (mmol/L)	4.6 [4.0, 5.9]	4.4 [3.5, 5.5]	0.041	4.6 [3.9, 5.5]	4.5 [3.5, 5.5]	0.233
Cr (umol/L)	60.0 [46.0, 73.0]	60.0 [49.0, 71.0]	0.790	58.0 [45.0, 72.0]	61.0 [51.5, 71.5]	0.131
SA (U/L)	156.5 [88.0, 365.0]	133.0 [69.0, 349.0]	0.478	158.0 [88.0, 365.0]	128.0 [60.5, 272.0]	0.471
SL (U/L)	585.0 [118.0, 1347.0]	499.0 [112.0, 1349.0]	0.736	578.0 [118.0, 1347.0]	341.0 [78.0, 949.0]	0.075
UA (U/L)	503.0 [243.0, 1066.0]	744.0 [336.0, 1843.0]	0.014	525.0 [263.0, 1066.0]	685.0 [279.0, 1339.0]	0.250

RAC, Revised Atlanta Classification; BISAP, Bedside Index for Severity in Acute Pancreatitis; MCTSI, Modified CT Severity index; Ca, Blood Calcium; PCT, Procalcitonin; ALT, Alanine aminotransferase; AST, Aspartate aminotransferase; ALB, Serum Albumin; UREA, Urea; Cr, Creatinine; SA, Serum Amylase; SL, Serum Lipase; UA, Urinary Amylase.

### 4.2 Comparative analysis of influencing factors post-treatment

Univariate logistic regression analysis indicated a significant reduction in CRP levels in the ITCWM group post-treatment (8.0 [3.3–33.5] in the ITCWM group vs. 17.8 [1.2–59.5] in the control group, *P* = 0.022). The median hospitalization duration in the ITCWM group was notably shorter at 11.5 days, in contrast to the 19.0 days in the control group, indicating the significant reduction of total treatment duration in the ITCWM group (*P* < 0.001) (Table 2). Multivariate analysis of demographic and clinical characteristics showed a generalized lower odds ratio (OR) in the ITCWM group, including CRP levels and hospitalization duration (OR for CRP levels = 0.976, 95% CI 0.953–0.999, *P* = 0.043; OR for hospitalization duration = 0.822, 95% CI 0.765–0.884, *P* < 0.001), suggesting the evident efficacy of TCM in reducing inflammation and hospitalization duration in mild to moderate AP (Table 3).

**TABLE 2:** Univariate comparison between the two groups.

Parameters (post-treatment)	Control group (61)	ITCWM group (120)	OR	95%CI	*P*
BISAP (points)	1.0 [0.3, 1.8]	0.0 [0.0, 1.0]	1.275	0.737–2.208	0.385
MCTSI (points)	1.0 [0.0, 2.0]	0.0 [0.0, 2.0]	1.118	0.779–1.604	0.546
APACHE II (points)	10.7 ± 2.1	10.9 ± 2.0	1.050	0.899–1.226	0.538
SOFA (points)	0.0 [0.0, 0.8]	0.0 [0.0, 1.0]	0.978	0.510–1.875	0.946
WBC (× 10^9^/L)	6.0 [5.0, 7.4]	6.5 [5.2, 7.5]	0.955	0.851–1.071	0.427
NEUT (%)	64.2 ± 13.7	59.8 ± 12.7	0.975	0.949–1.001	0.059
RBC (× 10^12^/L)	4.3 ± 0.7	4.4 ± 0.6	1.180	0.683–2.040	0.552
HGB (g/L)	131.5 ± 20.9	132.8 ± 18.3	1.004	0.986–1.022	0.695
Ca (mmol/L)	2.3 [1.9, 2.5]	2.3 [2.1, 2.4]	0.960	0.715–1.288	0.784
PLT (× 10^9^/L)	181.5 [100.3, 328.8]	193.0 [180.0, 280.0]	1.002	0.997–1.006	0.439
PCT (ng/mL)	0.1 [0.0, 2.3]	0.1 [0.0, 0.2]	0.548	0.222–1.352	0.192
CRP (mg/L)	17.8 [1.2, 59.5]	8.0 [3.3, 33.5]	0.974	0.944–1.005	0.022
ALT (U/L)	26.5 [16.3, 72.8]	23.0 [12.5, 37.5]	0.995	0.989–1.001	0.106
AST (U/L)	40.0 [21.5, 129.0]	26.0 [19.5, 29.5]	0.997	0.991–1.002	0.254
ALB (g/L)	36.4 ± 5.4	37.8 ± 4.3	1.066	0.971–1.171	0.177
SA (U/L)	78.0 [71.0, 83.5]	54.0 [34.5, 113.0]	1.011	0.998–1.004	0.649
SL (U/L)	139.0 [91.8, 1194.8]	94.0 [70.5, 927.5]	0.976	0.999–1.001	0.723
UA (U/L)	136.0 [44.8, 428.3]	381.0 [167.3, 607.6]	1.120	1.000–1.101	0.369
Hospital stays (days)	19.0 [18.1, 21.3]	11.5 [10.5, 14.0]	0.855	0.806–0.907	0.000

BISAP, Bedside Index for Severity in Acute Pancreatitis; MCTSI, Modified CT Severity Index; Ca, Blood Calcium; PLT, Platelets; PCT, Procalcitonin; ALT, Alanine aminotransferase; AST, Aspartate aminotransferase; ALB, Serum Albumin; SA, Serum Amylase; SL, Serum Lipase; UA, Urinary Amylase.

**TABLE 3:** Multivariate comparison between the two groups.

Parameters (post-treatment)	Control group (61)	ITCWM group (120)	OR	95%CI		*P*
	M (P_25_, P_75_)	M (P_25_, P_75_)		Lower	Upper	
CRP (mg/L)	17.8 [1.2, 59.5]	8.0 [3.3, 33.5]	0.976	0.953	0.999	0.043
Hospital stays (days)	19.0 [18.1, 21.3]	11.5 [10.5, 14.0]	0.822	0.765	0.884	0.000

### 4.3 Comparison of K-M survival curves for symptom relief duration

**FIGURE 2:**
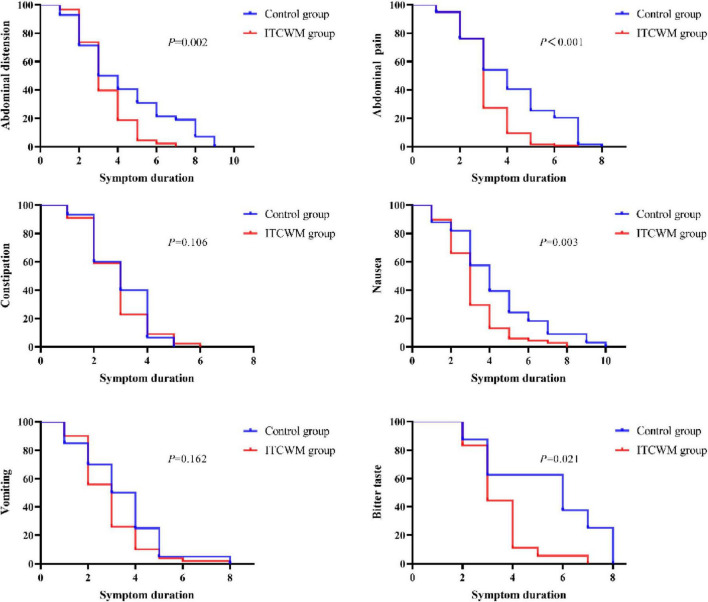
Comparison of K-M survival curves for symptom relief duration in the two groups.

The K-M survival analysis highlighted longer-term relief in symptoms like abdominal distension, abdominal pain, nausea and bitter taste in the ITCWM group (*P* = 0.000–0.021). However, the analysis found no significant difference in the relief of vomiting and constipation between the two groups (*P* > 0.05), as detailed in Figure 2.

### 4.4 Comparative analysis of post-treatment outcomes across the three groups

The study categorized 563 patients into three groups based on the inclusion of various TCM therapies in their treatment: control group (66 patients), OCM group (305 patients), and OCM+E group (192 patients). Following PSM, the groups were reorganized as control group (49), OCM group (274), and OCM+E group (131). Prior to matching, the baseline characteristics exhibited significant disparities among the three groups. For instance, the incidence of lipogenic pancreatitis was 35 (53.8%) in the control group, 193 (63.3%) in the OCM group, and 139 (72.0%) in the OCM+E group. The proportion of patients with mild acute pancreatitis (AP) was 38 (58.5%) in the control group, 241 (79.0%) in the OCM group, and 113 (58.5%) in the OCM+E group. Procalcitonin (PCT) levels (ng/mL) were 0.1 (0.1,0.2) in the control group, 0.1 (0.1, 0.2) in the OCM group, and 0.1 (0.1, 0.3) in the OCM+E group. Additionally, SA and SL levels were similarly imbalanced. Specifically, SA levels (U/L) were 158.0 (88.0,346.5) in the control group, 107.0 (61.5,236.5) in the OCM group, and 254.0 (108.0,571.5) in the OCM+E group. Similarly, SL levels (U/L) in the control group, OCM group, and OCM+E group were 592.0 (140.0,1251.6), 345.0 (91.0,870.5), and 943.0 (243.0,1924.0), respectively. Following matching, the baseline characteristics of the three groups were essentially balanced, as detailed in Supplementary Tables 3–4.

One-way ANOVA analysis revealed statistically significant superior therapeutic outcomes in the OCM+E group compared to the control group. Notably, neutrophil percentage (NEUT%) (59.5 vs. 74.1) was lower in the OCM+E group (*P* < 0.05), and blood calcium (Ca) (2.3 vs. 2.0), serum prealbumin (PA) (220.0 vs. 116.0) were notably higer in the OCM+E group, compared to the control group (*P* < 0.05). Additionally, the OCM+E group experienced a significantly reduced hospitalization duration compared to the control group (10.0 days vs. 19.0 days, *P* < 0.05), underscoring the efficacy of the combined treatment approach. Compared to the OCM group, AP patients in the OCM+E group had a significantly lower NEUT% (59.5 vs. 61.9, P < 0.05), and the reduction in hospitalization duration was more pronounced in the OCM+E group (Table 4).

**TABLE 4:** Univariate logistic regression analysis across the three groups.

Parameters (post-treatment)	Control group (49)	OCM group (274)	OCM+E group (131)	*P*
BISAP (points)	1.5 [0.0, 1.6]	0 [0.0, 0.0]	0 [0.0, 1.0]	0.068
MCTSI (points)	2.0 [2.0, 2.0]	0 [0.1, 1.5]	0 [0.3, 0.7]	0.150
APACHE II (points)	14.0 ± 1.4	10.4 ± 2.1	11.1 ± 2.6	0.242
SOFA(points)	0.0 [0.0, 1.0]	0 [0.0, 1.0]	0 [0.0, 0.0]	0.067
WBC (× 10^9^/L)	6.9 [5.9, 9.6]	6.8 [5.3, 8.8]	6.5 [5.7, 8.2]	0.096
NEUT%	74.1 ± 5.2	61.9 ± 15.0*	59.5 ± 12.3*^▲^	0.001
RBC (× 10^12^ /L)	4.9 [4.1, 13.9]	4.3 [4.0, 4.6]	4.3 [3.9, 4.5]	0.415
HGB (g/L)	95.5 ± 27.6	134.2 ± 8.8	121.0 ± 19.2	0.391
Ca (mmol/L)	2.0 ± 0.2	2.2 ± 0.1*	2.3 ± 0.1*	0.009
PLT (× 10^9^ /L)	258.0 [112.0, 343.0]	314.0 [150.8, 400.0]	233.0 [194.6, 306.6]	0.646
PCT (ng/mL)	0.7 [0.0, 1.3]	0.2 [0.1, 0.3]	0.1 [0.0, 0.2]	0.483
CRP (mg/L)	21.0 [2.0, 50.0]	19.5 [1.3, 52.1]	18.7 [1.4, 42.7]	0.146
ALT (U/L)	18.5 [8.7, 75.7]	20.0 [18.0, 30.4] *	16.0 [3.3, 53.3] *	0.041
AST (U/L)	24.0 [19.5, 87.5]	22.0 [18.9, 25.9]	26.0 [19.8, 39.6]	0.330
TP (g/L)	54.2 ± 0.2	61.0 ± 8.3	66.0 ± 3.8	0.992
ALB (g/L)	28.0 ± 0.8	35.5 ± 4.6	37.3 ± 3.0	0.282
TBIL (umol/L)	16.9 [17.0, 22.8]	16.3 [12.9, 21.3]	11.1 [9.4, 17.0]	0.095
DBIL (umol/L)	11.0 [5.6, 21.5]	8.0 [5.7, 10.5]	3.7 [3.1, 7.4]	0.380
PA (mg/L)	116.0 ± 24.0	184.4 ± 58.0*	220.0 ± 39.7*^▲^	0.001
UREA (mmol/L)	3.6 [2.4, 4.8]	4.4 [2.6, 5.1]	3.6 [2.8, 4.9]	0.684
Cr (umol/L)	53.0 ± 17.0	58.1 ± 15.0	61.7 ± 18.8	0.310
SA (U/L)	82.0 [79.0, 85.0]	78.0 [54.0, 83.0]	70.0 [62.0, 106.0]	0.795
SL (U/L)	157.5 [111.0, 204.0]	66.5 [56.0, 135.0]	97.0 [74.0, 120.0]	0.808
UA (U/L)	78.5 [16.0, 141.0]	561.5 [256.0, 1298.0]	367.0 [221.0,6 12.0]	0.777
Hospital duration (days)	19.0 [16.5, 22.0]	12.0 [8.0, 17.0] *	10.0 [7.0, 14.0]*^▲^	0.000

Comparison between the OCM+E group and the control group,

*P < 0.05; comparison between the OCM+E group and the OCM group,

▲*P* < 0.05; BISAP, Bedside Index for Severity in Acute Pancreatitis; MCTSI, Modified CT Severity Index; Ca, Blood Calcium; PLT, Platelets; PCT, Procalcitonin; ALT, Alanine aminotransferase; AST, Aspartate aminotransferase; TP, Total Protein; ALB, Serum Albumin; PA, Prealbumin; UREA, Urea; Cr, Creatinine; SA, Serum Amylase; SL, Serum Lipase; UA, Urine Amylase.

Upon incorporating demographic indicators, clinical characteristics, and variables with *P* < 0.05 into the multivariate logistic regression analysis, we identified NEUT%, PA levels (mg/L), and hospitalization duration as significant factors across the three groups. The OCM+E group had the shortest hospital duration (10.0 [OCM+E] vs. 12.0 [OCM] vs. 19.0 [control] d, *P* < 0.05). The NEUT% was lower and the PA level (mg/L) was higher in the OCM+E group compared to the other two groups (*P* < 0.05), as detailed in Table 5. These findings indicated the enhanced efficacy of TCM combined with Enema in mitigating inflammatory indicators, boosting immunity and shortening hospital duration, compared to Western medicine or TCM alone.

**TABLE 5:** Multivariate logistic regression analysis across the three groups.

Parameters (post-treatment)	Control group (49)	OCM group (274)	OCM+E group (131)	OR	95%CI		*P*
					Lower	Upper	
PA (mg/L)	116.0 ± 24.0	184.4 ± 58.0*	220.0 ± 39.7*^▲^	1.018	1.004	1.032	0.013
NEUT%	74.1 ± 5.2	61.9 ± 15.0*	59.5 ± 12.3*^▲^	0.857	0.811	0.945	0.001
Hospital duration (days)	19.0 [16.5, 22.0]	12.0 [8.0, 17.0] *	10.0 [7.0, 14.0]*^▲^	0.860	0.775	0.954	0.004

Comparison between the OCM+E group and the control group,

*P < 0.05; comparison between the OCM+E group and the OCM group,

▲*P* < 0.05; PA, Serum Prealbumin.

### 4.5 Comparative analysis of K-M survival curves for symptom relief duration across the three groups

**FIGURE 3:**
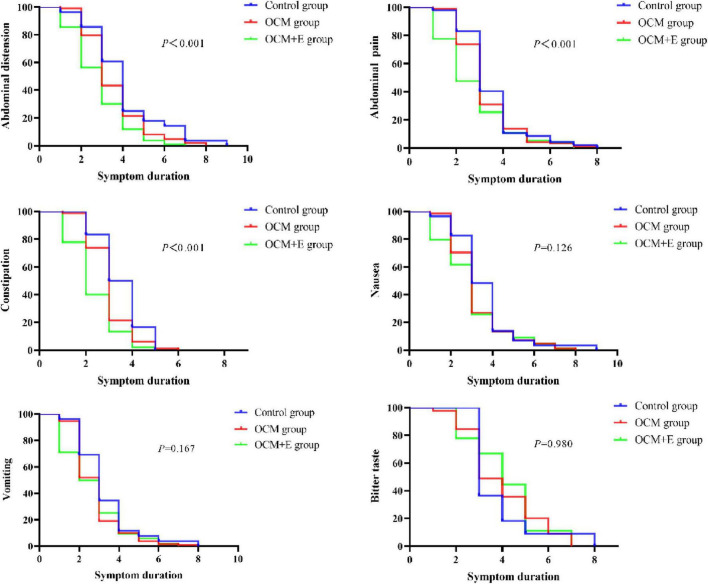
Comparison of K-M survival curves for the symptom relief duration across the three groups.

The comparative analysis revealed that the OCM+E group exhibited a significantly longer-term relief in abdominal distension, abdominal pain, and constipation, compared to the other two groups (*P* < 0.001). Conversely, the duration of symptoms, such as nausea, vomiting, and bitter taste, did not demonstrate statistically significant difference across the groups (*P* > 0.05) (Figure 3). This suggested that the integration of enema with TCM therapy might notably reduce the duration of certain symptoms in mild to moderate AP patients.

## 5 Discussion

TCM has demonstrated its potential in managing acute pancreatitis and individualized therapy, particularly in cases of mild to moderate AP. In TCM, AP is categorized into “stomach duct pain” and “spleen and heart pain”, originating from the spleen and involving the gallbladder, liver, and stomach. TCM attributes the mechanism of AP to “organ congestion and toxin accumulation” (15). Current TCM therapies for AP center on alleviating liver congestion, reducing heat and dampness, and ensuring smooth functioning of organs (15), all of which reflect the TCM principles of holistic adjustment and syndrome differentiation. Evidence has exhibited the ability of TCM to modulate the production of pro-inflammatory and anti-inflammatory cytokines in inflammatory responses, thus achieving such favorable outcomes as reduced hospitalization duration (7, 17). Due to its complex and capricious mechanism, AP can be effectively managed by adjusting the ratios of herbal components in a TCM formulation (18). TCM has shown its effectiveness in reducing both the incidence and recurrence of AP, thus enhancing the quality of life for patients (10). Prealbumin, a liver-synthesized serum biomarker, sensitively reflects the protein turnover and indicates the nutritional status in the body. A lower level of prealbumin represent a decline in overall health. TCM has been shown to elevate serum prealbumin (PA) levels, thereby promoting the recovery from AP (19). However, previous studies are primarily limited by small sample sizes, indicating a lower level of evidence-based medical support. Therefore, based on the real world, this study with a larger sample size, provides a stronger evidence of the reliable effectiveness of TCM in treating mild to moderate AP.

This study verifies that TCM posed positive impacts on inflammatory indicators, symptom and hospitalization duration in mild to moderate AP. Here, PSM was employed to ensure covariate balance between groups. After PSM, TCM exhibited significant effectiveness in improving inflammatory indicators, curtailing hospitalization duration, and diminishing symptom duration in mild to moderate AP. These results, aligning with those in prior research, further prove the therapeutic potential of TCM in coping with AP. Additionally, the study highlighted the efficacy of herbal enema, a characteristic TCM therapy, in enhancing laboratory indicators and clinical symptoms in AP. Notably, the safety of combining TCM and enema therapy was commendable (8, 9, 20). Extensive clinical research has demonstrated that rhubarb enema therapy is effective in treating acute pancreatitis (AP) (9, 21–23). Especially, using a 200 ml solution of 30 g raw rhubarb infusion for jejunal irrigation has shown excellent therapeutic results for AP (9, 21). Other studies indicate that raw rhubarb enema can reduce serum inflammatory cytokines, C-reactive protein (CRP), and endotoxin levels in AP patients, thereby alleviating systemic inflammatory stress responses (21, 24, 25). It has been proven beneficial in protecting pancreatic function and delaying disease progression (9, 10, 18). Furthermore, in patients suffering from severe abdominal symptoms, such as distension, pain, and constipation, herbal enema showed efficacy in stimulating gastrointestinal motility and boosting mucosal absorption. Additionally, the herbal medicine could serve as lubricants to aid bowel movements and thus reduce intra-abdominal pressure due to accumulated intestinal content (26–28). Moreover, herbal enema avoids gastrointestinal mucosal irritation and liver first-pass effects. To further assess the effectiveness of TCM combined with enema for AP, we subdivided the ITCWM group into TCM+ enema and TCM alone. Our findings indicated a notable superiority of the TCM+ enema over the other two therapies in mitigating inflammatory indicators, shortening symptom and hospitalization durations in AP patients. Additionally, this combination therapy raised the prealbumin level, consequently enhancing immune response.

Nevertheless, there remain limitations in the present study, such as the sample size inadequate, particularly within the control group, which might have limited the robustness of our statistical conclusions. Additionally, the lack of endpoint events impeded precise statistical analysis. Moreover, our patients mainly hailed from Henan, China, potentially limiting the generalizability of our findings. Despite using PSM to balance covariates between the groups, the retrospective nature of this study limited its validity due to non-randomization and other potential biases, including selection, informational, and recall biases. Consequently, the evidence level provided by our study was insufficiently persuasive. Prospective studies, particularly rigorously homogenized randomized controlled trials, are recommended to provide more robust substantiation.

## Data Availability

The original contributions presented in the study are included in the article/Supplementary material, further inquiries can be directed to the corresponding authors.

## Footnotes

1
https://fast.statsape.com
