# Supplementary material for: Efficacy of integrated traditional Chinese and western medicine in managing mild-moderate acute pancreatitis: a real-world clinical perspective analysis
Source: Front Med (Lausanne). 2024 Oct 30;11:1429546. doi: 10.3389/fmed.2024.1429546 (PMC11566454; doi:10.3389/fmed.2024.1429546)
Supplement: Supplementary file 1 [file Table_1.docx]

**Supplementary Table1:** TCM Classification and Treatment of Acute Pancreatitis

| Syndrome | Liver depression and qi stagnation | Liver and gallbladder dampness and heat | Chest binding and interior excess | Blood stasis(toxin) binding with hot |
| --- | --- | --- | --- | --- |
| Main symptoms | (1) Right and middle upper abdominal pain, (2)distension and pain of the bilateral hypochondrium relieved with a fart. | (1) Bilateral flank rib distension pain, (2) bitter taste and nausea. | (1)Hard and full of pain in the chest, hypochondrium, and upper abdomen, even with tenderness, (2) fullness and discomfort in chest and hypochondrium | (1) Stabbing abdominal pain refused to press without transferred and (2) bleeding, skin bruising, and freckle. |
| Secondary symptoms | (1)Depression, irritability, and sighing, (2)nausea and vomiting, (3) belching and hiccups, (4) constipation. | (1) Eyes and body with jaundice, (2) irregular stool, (3) less and yellow urine, (4) tired with poor appetite | (1) Alternating chill and fever, (2) vexation and vomiting, (3) difficulty and pain in micturition with short red urine, (4) constipation | (1) Fever especially in night, (2) short red urine, (3) dry and knotted stool, (4) a palpable mass in the abdomen |
| Tongue and pulse | Reddish tongue with thin white or thin yellow coat and wiry and tight or wiry and rapid pulse, especially on left Guan. | Red tongue with yellow greasy or thin yellow coat and wiry and rapid or wiry, slippery, and rapid pulse, especially on left Guan | Red tongue with yellow greasy or yellow thick and dry coat and slippery and rapid or deep tight or deep, rapid, and powerful pulse | Red tongue or ecchymosis and wiry and rapid or hesitant pulse |
| Diagnosis | Two main symptoms complicated with at least one secondary symptom. With atypical symptoms, tongue and pulsation, as well as physic and laboratory examinations, should be considered | | | |
| Treatment principle | Dispersing stagnated liver Qi | Clearing away heat and dampness in liver and gallbladder | Removing stasis by purgation, regulating Qi, and activating blood | Clearing heat and purging fire, removing blood stasis, and communicating with the Fu organs |
| Prescription | The modification of Chaihu Shugan Powder (Jing Yue Book) and Qing Yi Decoction | The modification of Yinchenhao Decoction (Treatise on Febrile Diseases) combined with LongDanxiegan Decoction (Collection of Prescriptions with Notes) or Qingyi Decoction | The modification of Qingyi Decoction and Daxianxiong Decotion (Treatise on Febrile Diseases) | The modification of Xiexin Decoction (Treatise on Febrile Diseases) or Dahuangmudan Decoction (Synopsis of the Golden Chamber) combined with Gexiazhuyu Decoction (Correcting Mistakes by Medical Forests) |

| **Supplementary Table2:** Symptoms of two groups after PSM analysis | | | | |
| --- | --- | --- | --- | --- |
| Symptom[n (%)] | Control group(61) | TCM group(120) | $x^{2}$ | *P* |
| Abdominal distension | 43(70.5) | 91(75.8) | .355 | .552 |
| Abdominal pain | 61(100) | 117(97.5) | .396 | .529 |
| Constipation | 16(26.7) | 45(37.5) | 1.640 | .200 |
| Nausea | 34(55.7) | 69(57.5) | .005 | .946 |
| Vomiting | 21(34.4) | 51(42.5) | .789 | .374 |
| Bitter taste | 8(13.1) | 18(15.0) | .014 | .906 |
| Thirst | 15(24.6) | 26(21.7) | .066 | .798 |
| Fever | 1(1.6) | 8(6.7) | 1.230 | .267 |

| **Supplementary Table3**: Comparison of basic characteristics across the three groups before and after PSM | | | | | | | | |
| --- | --- | --- | --- | --- | --- | --- | --- | --- |
| Items | Before Matching | | | | After Matching | | | |
|  | Control group(66) | OCM group(305) | OCM+E group(192) | *P* | Control group(49) | OCM group(274) | OCM+E group(131) | *P* |
| Age (years) | 39.0 [31.0,55.0] | 41.0 [34.5,50.0] | 37.0 [33.0,48.0] | .400 | 39.0 [38.6,48.2] | 41.0 [42.6,46.1] | 39.0 [40.0,45.0] | .486 |
| Gender male[n (%)] | 42 (64.6) | 223 (73.1) | 135 (69.9) | .361 | 31(63.3) | 195 (71.2) | 94（71.8） | .505 |
| Weight（kg） | 71.3±13.0 | 74.6±13.1 | 72.7±13.4 | .103 | 71.0±12.7 | 74.0±12.7 | 72.7±13.7 | .281 |
| Pathogenesis[n (%)] |  |  |  |  |  |  |  |  |
| Biliary | 20 (30.8) | 113 (37.0) | 55 (28.5) | .126 | 17 (34.7) | 96 (35.0) | 42（32.1） | .835 |
| Lipogenic | 35 (53.8) | 193 (63.3) | 139 (72.0) | .017 | 28(57.1) | 176 (64.2) | 92（70.2） | .222 |
| Alcoholic | 1 (1.5) | 1 (0.3) | 4 (2.1) | .130 | 1 (2.0) | 1 (0.4) | 1 (0.8) | .684 |
| Smoke | 17 (26.2) | 69 (22.6) | 57 (29.5) | .218 | 12 (24.5) | 62 (22.6) | 40 (30.5) | .228 |
| Drink | 19 (29.2) | 84 (27.5) | 67 (34.7) | .228 | 14 (28.6) | 74 (27.0) | 47 (35.9) | .186 |
| Co-morbidities [n (%)] |  |  |  |  |  |  |  |  |
| Hypertension | 9 (13.8) | 64 (21.0) | 45 (23.3) | .275 | 6 (12.2) | 56 (20.4) | 31 (23.7) | .244 |
| Diabetes | 12 (18.5) | 60 (19.7) | 44 (22.8) | .625 | 6 (12.2) | 57 (20.8) | 32 (24.4) | .198 |
| RAC(mild)[n (%)] | 38（58.5） | 241（79.0） | 113（58.5） | .000 | 31（63.3） | 210（76.6） | 89（67.9） | .151 |
| BISAP（points） | 0.0 [0.0,1.0] | 0.0 [0.0,1.0] | 1.0 [0.0,2.0] | .000 | 0.0 [0.3,0.7] | 0.0 [0.3,0.4] | 1.0 [0.6,0.9] | .000 |
| MCTSI（points） | 2.3±0.9 | 2.1±0.6 | 2.6±1.0 | .000 | 2.2±0.7 | 2.2±0.6 | 2.3±0.8 | .052 |
| APACHE II（points） | 13.4±2.8 | 13.1±2.5 | 13.5±3.0 | .205 | 13.2±2.7 | 13.2±2.5 | 13.1±2.8 | .967 |
| SOFA(points) | 0.0 [0.0,1.0] | 0.0 [0.0,1.0] | 0.0 [0.0,1.0] | 0.294 | 0.0 [0.3,0.8] | 0.0 [0.5,0.6] | 0.0 [0.5,0.7] | 0.859 |
| WBC(×10^9^/L) | 9.4 [5.7,13.1] | 8.2 [5.9，11.3] | 11.8[9.2,14.9] | .000 | 9.5 [8.6，11.2] | 8.7 [8.9,9.9] | 10.8 [10.3,11.8] | .001 |
| NEUT(%) | 77.6±12.9 | 74.6±12.2 | 81.9±12.3 | .000 | 78.3±13.0 | 75.3±12.2 | 79.1±13.6 | .012 |
| RBC(×10^12^ /L) | 4.7 [4.1,5.1] | 4.7 [4.3,5.0] | 4.7 [4.3,5.2] | .031 | 4.7 [1.4,14.1] | 4.7 [4.6,4.7] | 4.7 [4.5,4.8] | .020 |
| HGB(g/L) | 139.4±25.2 | 142.4±21.8 | 144.5±26.5 | .301 | 139.3±25.0 | 142.5±21.5 | 142.9±27.2 | .637 |
| Ca (mmol/L) | 2.3±0.2 | 2.3±0.2 | 2.3±0.2 | .007 | 2.3±0.2 | 2.3±0.2 | 2.3±0.2 | .297 |
| PLT(×10^9^/L) | 216.3±68.6 | 230.5±78.9 | 229.0±76.6 | .402 | 222.8±59.6 | 228.4±78.7 | 224.3±73.1 | .812 |
| PCT(ng/mL) | 0.1 [0.1,0.2] | 0.1 [0.1,0.2] | 0.1 [0.1,0.3] | .020 | 0.1 [0.1,0.8] | 0.1 [0.2,0.3] | 0.1 [0.1,1.7] | .050 |
| CRP(mg/L) | 11.0 [3.2,34.2] | 18.0[6.5,54.0] | 23.0 [7.1,70.3] | .007 | 11.9 [17.6,42.3] | 18.0 [34.8,46.1] | 23.0 [35.8,52.7] | .201 |
| ALT(U/L) | 28.0 [16.5,71.5] | 28.0 [17.0,61.5] | 32.0 [18.5,63.0] | .715 | 28.0 [38.2,94.1] | 24.5 [52.7,95.1] | 31.0 [41.4,82.5] | .760 |
| AST(U/L) | 26.0 [19.0,64.8] | 29.0 [20.0,48.0] | 27.0 [20.0,47.5] | .734 | 26.0 [42.3,104.6] | 26.5 [33.6,153.5] | 24.9 [41.0,78.3] | .717 |
| ALB(g/L) | 41.7 [38.7,43.7] | 40.5 [37.6,42.8] | 40.7[37.0,43.4] | .277 | 41.7 [40.1,42.2] | 40.5 [39.,40.9] | 40.8 [39.8,41.4] | .548 |
| UREA(mmol/L) | 5.1±2.6 | 4.6±2.3 | 5.0±2.8 | .148 | 5.1±2.9 | 4.7±2.3 | 4.8±1.7 | .494 |
| Cr (umol/L) | 59.3 [47.5，72.0] | 59.0 [50.0，70.0] | 61.0 [50.0，71.0] | .105 | 59.3 [38.4,122.4] | 58.5 [58.1,63.3] | 60.0 [57.4,63.5] | .140 |
| SA (U/L) | 158.0 [88.0,346.5] | 107.0 [61.5,236.5] | 254.0 [108.0,571.5] | .001 | 162.0 [88.0，365.0] | 114.0 [64.0,268.0] | 196.0 [101.5,412.5] | .143 |
| SL (U/L) | 592.0 [140.0,1251.6] | 345.0 [91.0,870.5] | 943.0 [243.0,1924.0] | .000 | 641.0 [167.0，1346.0] | 694.4 [97.0，986.0] | 758.0 [190.0，1711.5] | .726 |
| UA (U/L) | 681.0 [226.0,2101.5] | 740.8 [488.3,1540.7] | 1099.7 [726.4,3258.3] | .000 | 1066.0 [599.0,1877.0] | 761.0 [492.6,1653.6] | 1261.0 [643.2,3082.2] | . 729 |

(BISAP: Bedside Index for Severity in Acute Pancreatitis; MCTSI: Modified CT severity index; Ca: blood calcium; PLT: platelets; PCT: procalcitonin; ALB: serum albumin; UREA: urea; Cr: creatinine; SA: serum amylase; SL: serum lipase; UA: urine amylase).

| **Supplementary Table4**：Symptoms of three groups after PSM analysis | | | | | |
| --- | --- | --- | --- | --- | --- |
| Symptom[n (%)] | Control group  (49) | OCM group  (274) | OCM+E group  (131) | $x^{2}$ | *P* |
| Abdominal distension | 35(10.0) | 205(58.7) | 109(31.2) | 4.423 | .110 |
| Abdominal pain | 49(10.9) | 271(60.4) | 129(28.7) | .763 | .683 |
| Constipation | 15(10.5) | 72(50.3) | 56(39.2) | 11.132 | .004 |
| Nausea | 26(9.1) | 170(59.2) | 91(31.7) | 4.535 | .104 |
| Vomiting | 18(8.6) | 125(59.5) | 67(31.9) | 3.091 | .213 |
| Bitter taste | 7(11.1) | 46(73.0) | 10(15.9) | 6.223 | .045 |
| Thirst | 14(15.2) | 62(67.4) | 16(17.4) | 8.295 | .116 |
| Fever | 1(4.2) | 13(54.2) | 10(41.7) | 2.633 | .268 |
